# Innate and Adaptive Immune Responses to Herpes Simplex Virus

**DOI:** 10.3390/v1030979

**Published:** 2009-11-18

**Authors:** Tracy Chew, Kathryne E. Taylor, Karen L. Mossman

**Affiliations:** 1 Department of Pathology and Molecular Medicine, McMaster University, Hamilton, Ontario, Canada; E-Mail: chewt@mcmaster.ca; 2 Department of Biochemistry and Biomedical Sciences, McMaster University, Hamilton, Ontario, Canada; E-Mail: tayloke@mcmaster.ca

**Keywords:** Herpes Simplex virus (HSV), innate immunity, antiviral signaling, type I interferon (IFN), Natural killer (NK) cells, plasmacytoid dendritic cells (pDCs), adaptive immunity

## Abstract

Immune responses against HSV-1 and HSV-2 are complex and involve a delicate interplay between innate signaling pathways and adaptive immune responses. The innate response to HSV involves the induction of type I IFN, whose role in protection against disease is well characterized *in vitro* and *in vivo*. Cell types such as NK cells and pDCs contribute to innate anti-HSV responses *in vivo*. Finally, the adaptive response includes both humoral and cellular components that play important roles in antiviral control and latency. This review summarizes the innate and adaptive effectors that contribute to susceptibility, immune control and pathogenesis of HSV, and highlights the delicate interplay between these two important arms of immunity.

## 1. Introduction

Herpes simplex virus (HSV) is a highly successful human pathogen belonging to the family *Herpesviridae.* Two serotypes of the alpha herpesviruses, HSV-1 and HSV-2, are the causative agents of oral and genital herpes, whose lifelong infections are highly prevalent in North America and worldwide. The immune response to these pathogens is complex and multifactorial; likewise, these ancient viruses have multiple viral evasion mechanisms that have secured their evolutionary success. Therefore, the study of anti-HSV immune responses as well as the corresponding viral countermeasures is important to our understanding of antiviral immunity and viral pathogenesis in general.

The immune response against HSV involves both innate and adaptive immune mechanisms. The innate antiviral response is thought to play a pivotal role in determining the outcome of an HSV infection. Accordingly, the production of type I interferon (IFN), comprised largely of IFNα and IFNβ, has been linked to protection against disease in both mouse models and in human studies [1–9]. In addition, multiple cell types have been shown to contribute to the innate immune responses to HSV *in vivo*. Among the most important of these cell types are natural killer (NK) cells, whose role in anti-HSV immunity involves cytokine production, recognition, and killing of virally infected cells, and plasmacytoid dendritic cells (pDCs), whose primary role involves type I IFN production *in vivo* [10–14].

In addition, the adaptive immune response has been shown to play important roles in disease progression, latency and control of virus spread. Neutralizing antibody levels have been negatively correlated with disease severity [15–17]; however, conflicting studies have failed to identify a precise role for the antibody-mediated response to HSV *in vivo* in murine models of infection [16,18]. The cellular response is highly involved in antiviral defense, with CD8+ T cells playing an important role in this process, largely through the production of IFNγ [19–22]. A minor role for CD4+ T cells has been described, with these cells providing some degree of protection in the absence of other immune effectors [20–23].

In this review, we summarize the current findings that characterize the role of both innate and adaptive immune responses against HSV. We first discuss the innate antiviral signaling pathways that lead to type I IFN production, as well as the corresponding downstream effector pathways. Second, we describe the role of NK cells and pDCs in the establishment of innate immunity *in vivo*. Finally, we discuss the role of both humoral and cell-mediated immunity as it contributes to protection, viral control and reactivation.

## 2. The role of type I interferons *in vivo*

The type I IFNs are key factors involved in the host defense against HSV. Interest in the involvement of these cytokines in the resistance to HSV began with the observation that different strains of mice show significant variation in their susceptibility to infection with HSV-1, with C57BL/6 mice demonstrating a much higher resistance compared to the A and BALB/c strains of mice [3]. Many groups have suggested that this observation could be attributed to differences between mouse strains in their ability to mount a rapid type I IFN response [2,4,9,24], although alternative explanations were proposed [25–31]. Recent studies, however, have more clearly confirmed the importance of type I IFN in HSV resistance. For example, based on experiments using SCID mice, which lack lymphocytes but have normal innate immune responses [32], Halford *et al.* have suggested that the difference between mouse strains historically categorized as “resistant” (C57BL/6) or “susceptible” (BALB/c mice) can be explained by the increased ability of C57BL/6 to develop a rapid type I IFN response [33]. Studies using IFNα/β receptor knockout mice have further substantiated this observation, as increased viral replication and pathogenesis as well as decreased survival have been shown in these mice after genital infection with HSV-2 [34–36] and ocular or footpad challenge with HSV-1 [37,38]. Finally, Vollstedt *et al.* have demonstrated that mice lacking type I IFN signaling have strongly increased susceptibility to intraperitoneal inoculation with HSV-1, while mice deficient in the recombination activating gene (RAG), which have intact IFN responses but no mature T or B cells, showed resistance to HSV-1 infection [6]. The influential function of the type I IFNs in the defense against HSV has been corroborated by reports that human patients suffering from unusually severe infections with HSV, such as herpes simplex encephalitis, often have defects in type I IFN signaling [39–41]. These studies highlight the significant role of innate immunity, particularly of the type I IFN response, in resistance to HSV infection.

## 3. Recognition of HSV and activation of type I IFN pathways

The molecular mechanisms leading to the induction of antiviral immunity against viruses are multifaceted, and involve multiple cellular recognition events. The detection of viral components involves multiple pattern recognition receptors (PRRs) and results in signal transduction pathways that ultimately lead to the production of type I IFN.

The type I IFN signaling pathway begins with the recognition of viral protein or nucleic acid. Recognition of viral ligands by PRRs leads to downstream signaling that ultimately converges on the activation of either Tank binding kinase 1 (TBK-1) or Inhibitor of NFκB epsilon (IKKɛ). The activation of these kinases leads to activation of IFN regulatory factor 3 (IRF-3), a transcription factor whose role in the expression of type I IFN and downstream pathways is thought to be of paramount importance. Activation of IRF-3, along with other transcription factors including NFκB and ATF/cJun, leads to the production and secretion of type I IFN. Autocrine and paracrine signaling via type I IFN occurs through a JAK/STAT pathway and leads to the production of effector molecules known as IFN stimulated genes (ISGs), whose collective role is to limit viral replication and spread through the establishment of an antiviral state.

### 3.1. Cellular sensors of HSV

A wide variety of viral products serve as triggers for the cellular recognition of HSV infection, including nucleic acids and lipoproteins. The nucleic acid sensors can be categorized based on both localization and nucleic acid structure, and include the scavenger receptors, the retinoic acid inducible gene I (RIG-I)-like receptors (RLRs), the Toll-like receptors (TLRs), the Nod-like receptors (NLRs) and DNA-dependent activator of IRFs (DAI) [42–47]. The viral protein sensors include the TLRs, which have been shown to recognize viral lipopeptides [48,49].

The major trigger for cellular recognition of a viral infection appears to be viral nucleic acid, which can be recognized at multiple cellular locations. Extracellular dsRNA is detected at the cell surface by the class A scavenger receptors [46,48]. Recent data suggest that these membrane-bound receptors recognize extracellular dsRNA and serve as chaperones to bring viral dsRNA into the cell for cytosolic recognition. In the endosomal compartment, TLR3 serves as a receptor for viral dsRNA and TLR9 recognizes CpG-rich DNA [14,42,50]. In the cytoplasmic compartment, RIG-I and melanoma differentiation associated gene 5 (MDA5) recognize viral dsRNA, with both redundancy and specificity with respect to both nucleic acid length and structure [51,52]. The cytoplasmic DNA sensors include DAI as well as the inflammasome activators Nalp3 and absent in melanoma 2 (AIM2) [43,44,47,53]. A recent paper by Chiu *et al.* highlights the redundancy and overlapping nature of these sensors, as viral DNA processing by RNA polymerase III yields ssRNA with 5′ triphosphate, which serves as a ligand for RIG-I [54].

For HSV in particular, a number of these receptors have been implicated specifically in the recognition of viral nucleic acids or proteins. For example, Wang *et al.* have suggested a direct role for DAI in sensing HSV infection, due to the ability of the DAI inhibitor adenosine deaminase acting on RNA 1 (ADAR1) to suppress HSV-1-mediated IFNβ gene expression [55]. In addition, HSV-1 infection activates the inflammasome, presumably through the nucleic acid sensor Nalp3 [47]. RIG-I has been recently implicated in the recognition of HSV in coordination with TLR9 [56,57].

TLRs have been shown to recognize both nucleic acid and protein derived from HSV [14,48,49,58,59]. In mice, four TLRs have been found to play a role in the resistance to HSV-1: TLR3, TLR9, and the TLR2/6 heterodimer, which recognize dsRNA, CpG-rich DNA and lipopeptides, respectively [48,49,56,59,60]. HSV-1 infection via intranasal delivery causes 100% mortality in mice deficient in the TLR adaptor protein MyD88, suggesting that innate control via TLRs is important in limiting HSV-1 infection [61]. Indeed, it has been shown *in vivo* that HSV-1-mediated production of IFN requires TLR9 and MyD88 [59,62].

The importance and downstream sequelae of TLR recognition of HSV is complex and context-dependent. TLR9 appears to play only a minor role in the defense against HSV despite its requirement for IFN production by pDCs. In the absence of TLR9, there was no change in either viral load in the nervous system or overall survival following infection with HSV-1 [59,62]. Furthermore, Kurt-Jones *et al.* have demonstrated that TLR2 mediates the production of inflammatory cytokines in response to HSV-1 infection, leading to the development of lethal viral encephalitis [49]. Taken together, this evidence suggests that while the precise role of TLRs in the innate response to HSV *in vivo* are not fully understood, they appear to have a role in mediating both immune protection as well as immune pathology. Not surprisingly, there has been a great deal of recent interest in using TLR ligands to treat or prevent HSV infection. A number of groups have revealed that mucosal delivery of ligands for TLR3 and TLR9, but not TLR2 or TLR4, are protective against genital infection with HSV-2 [35,63–68]. Similarly, intranasal application of a ligand for TLR3, but not for TLR4 or TLR9, protected against HSV encephalitis in mice [69]. The therapeutic approach of using TLR ligands to treat HSV is an area that requires further investigation, since the downstream consequences of TLR engagement *in vivo* remain to be clarified.

While viral nucleic acid and protein serve as potent triggers of antiviral signaling, increasing evidence suggests a role for the viral entry event in innate immune responses to virus. IRF-3 activation and ISG induction occur in response to virus particle entry in the absence of replication [70–76]. Data from our laboratory has demonstrated that the cellular response to virus particle entry requires IRF-3 and induces ISGs in the absence of IFN production and viral replication in primary fibroblasts [71]. In this pathway, NFκB is not activated and IRF-3 is a crucial component, as siRNA-mediated knockdown of IRF-3 abrogates this response [73,74]. This immediate early response does not depend on RIG-I, or any of the known TLRs [74]. Given the multifaceted nature of viral entry, cellular changes associated with this fusion event may serve as a danger signal for induction of an antiviral response. Indeed, generic stress signals such as ATP hydrolysis and changes to intracellular potassium ion concentration are sufficient triggers to induce an antiviral response by activating the Nalp3 inflammasome [77–79], suggesting that antiviral signaling may occur by virus recognition through danger associated patterns rather than by classical pathogen associated molecular patterns (PAMPs). The specific cellular stress and the corresponding danger recognition receptor involved in virus entry remain unknown, but have important implications in innate antiviral signaling.

### 3.2. Signal transduction pathways leading to type I IFN production

Following virus recognition by various cellular receptors, signal transduction events lead to the expression of type I IFNs. The cytosolic RIG-I, MDA5 and DAI pathways converge at the mitochondrial surface. Here, the adaptor protein MAVS associates with the nucleic acid sensor and recruits components of the antiviral signaling complex including TBK-1/IKKɛ, the adaptor protein mediator of IRF-3 activation (MITA, also known as STING), and IRF-3 [58,80–82]. The cellular components involved downstream of scavenger receptor binding and following recognition of virus entry are less well characterized, but like the other known sensors, the pathway appears to converge at the activation of the kinases TBK-1/IKKe [71,74,80,81]. TLR3-mediated recognition of virus also leads to activation of TBK-1 and IRF-3 in a TRIF-dependent manner, independent of MAVS or MITA [42]. Dimerization and activation of TBK-1 at the mitochondrial surface leads to activation of IRF-3 [80,81,83], resulting in IFNβ transcription [84–89]. Not surprisingly, the HSV-1 immediate early gene product ICP0 has been shown to block IRF-3 activity and prevent IFNβ transcription, highlighting the importance of IRF-3 in the antiviral cascade [90–92].

Type I IFN is thought to act autocrinely and paracrinely to generate a positive feedback loop that results in the induction of over 2000 ISGs. IFNβ signaling occurs through a JAK/STAT mechanism that leads to the induction of IFNα subspecies as well as ISGs such as IRF-7. IRF-7 is thought to be a master regulator of type I IFN production, as its activation is important to induce the full range of ISGs involved in eliciting an antiviral state during the late or amplification phase of the antiviral response, particularly in plasmacytoid dendritic cells (pDCs) [93].

### 3.3. Downstream effector pathways

The antiviral state is established by the collective activity of ISGs. These effectors have multiple distinct and overlapping functions, including inhibition of viral protein expression, apoptosis, and recruitment of immune cells to sites of infection [94–98]. These effector proteins are particularly important in the establishment of antiviral immunity against HSV, and their role is highlighted by the multiple viral countermeasures employed by HSV against these genes (reviewed in [99]). Promyelocytic leukemia (PML) protein forms ND10 nuclear structures [100], which have been implicated in a number of cellular antiviral processes, including the induction of apoptosis and major histocompatibility complex (MHC) class I antigen presentation [101,102]. Its importance in the innate response against HSV is highlighted by the fact that ICP0 disrupts these nuclear bodies, and knockdown of PML by shRNA increases viral gene expression and replication in primary fibroblasts [103,104]. Protein kinase R (PKR) is an IFN-inducible dsRNA-binding protein that phosphorylates and inhibits the activity of the eukaryotic translation initiation factor eIF2α, thus preventing viral gene expression [105]. Accordingly, mice deficient in PKR have increased susceptibility to HSV-1 and HSV-2 [106]. In fact, it has been suggested that the PKR pathway is key to the ability of IFNα1 to mediated defense against HSV-2 [107]. Two HSV-1 encoded genes, ICP34.5 and Us11, counteract PKR activity by dephosphorylating eIF2α and by competing for dsRNA binding, respectively [108,109]. Finally, the HSV-encoded viral host shutoff (vhs) protein inhibits host gene expression and decreases type I IFN production [110], attesting to the importance of this signaling pathway.

## 4. Cell type specific contributions to innate immunity against HSV

*In vivo*, several cell types contribute to the innate immune response against HSV. NK cells play an important role in both cytokine production and in recognition and killing of virally infected cells. In addition, the role of pDCs in the anti-HSV response has been described. Indeed, these cells were originally identified as natural IFN producing cells, as they are the major producers of IFNα *in vivo*.

### 4.1. NK cells

NK cells are important cellular components of the innate immune response. They have two major roles: to kill tumor or infected cells and to produce cytokines such as IFNγ [10,12,13]. The binding of ligands to either NK inhibitory or activating receptors determines the effector function of these cells [111,112]. Inhibitory receptors recognize MHC class I proteins on the uninfected cells and prevent NK cell activation [11]. Virus-mediated downregulation of MHC is a common mechanism of viral immune escape, as it prevents antigen presentation and adaptive antiviral responses. However, downregulation of MHC causes a decrease in inhibitory signals to the NK cell, leading to its activation and the lysis of the target cell (reviewed in [113]). Indeed, the HSV immediate-early protein ICP47 downregulates MHC class I by binding the cellular antigen transporter TAP1, preventing the transport of MHC molecules to the cell surface [114]. While this allows the virus to evade detection by CD8+ T cells, the absence of MHC increases the susceptibility of infected cells to NK lysis [115–117]. Importantly, NK cells also contain activation receptors which may directly detect viral proteins, leading to viral clearance [118].

Attempts to define a role for NK cells against HSV using depletion studies have yielded conflicting results. A number of studies have suggested that NK cell depletion via anti-NK1.1 or anti-asialo-GM1 antibodies increases the susceptibility of mice to ocular, genital, cutaneous and intravascular challenge with HSV [119–125]. In contrast, others have found that the depletion of NK cells with anti-asialo-GM1 antibodies does not impair the defense against intraperitoneal or footpad challenge with HSV [126–128]. In all cases, however, the findings of these studies are limited by the fact that asialo-GM1 expression is found on cell types other than NK cells, including epidermal dendritic cells, macrophages and activated T cells [129–132], while the NK1.1 antigen is not expressed on all NK cells, and can also be found on virus-specific activated T cells [133,134]. These discrepancies were underscored in an elegant study by Halford *et al*, who found that treatment with anti-asialo GM1 antibodies increased HSV-1 titers after ocular inoculation in BALB/c mice but not in SCID mice [133]. This observation implies that the anti-asialo-GM1 treatment exerted its effects on a lymphocyte population not present in SCID mice as opposed to NK cells, which are present in both strains.

In support of the involvement of NK cells in the defense against HSV, adoptive transfer of NK 1.1^+^ and asialo GM-1^+^ cells restores resistance to HSV, while transfer of cells from beige mice, which are deficient in NK cells, does not [135]. In another study, mice lacking both NK cells and T cells developed severe CNS infection leading to mortality after intranasal infection with HSV-1, while mice deficient only in T cells did not [136]. It has also been established that mice deficient in CCR5 expression suffer from increased susceptibility to genital challenge with HSV-2 [125]. These mice display no differences in T cell or total leukocyte recruitment, but decreased NK cell recruitment and activity was observed. In addition, Ashkar *et al.* compared the susceptibility to intravaginal challenge with HSV-2 in a number of mouse strains and found that the two most susceptible strains lack NK and NKT cells [63]. IL-15^–/–^ mice, which lack NK and NKT cells [137], showed higher susceptibility than mice lacking both T and B cells [63]. However, these findings are somewhat confounded by the fact that IL-15 is capable of producing antiviral effects in the absence of NK and NKT cells [138]. Finally, Grubor-Bauk *et al.* used mice deficient in CD1, which is required for the development of NKT cells, to show that this cell subset is important for the control of viral spread and morbidity in the HSV-1 zosteriform model [139].

In contrast, several studies have suggested that NK cells are not essential to the defense against HSV. For instance, despite a correlation between virulence of HSV strains and their ability to activate NK cells, treatments that strongly increased NK cell activity were unable to protect mice from HSV pathology and mortality [140]. In another study, Vollstedt *et al.* compared *rag2^–^/^–^* mice (which are deficient in T and B cells) and *rag2^–^/^–^* *γ_c_* *^–^/^–^* mice (which have an additional deficiency in NK cells) and found that NK cells are not required for resistance to HSV-1 [6]. Finally, mice overexpressing IL-15 were unable to survive HSV challenge, despite having higher numbers of NK cells and lower viral titers early in the infection [141].

The variation in the role of NK cells in the defense against HSV may be due, in part, to differences between the strains of mice and viruses used, as well as the mechanism of NK cell depletion and the route of inoculation. It is also difficult to assess whether studies in mice are applicable to humans. For example, although ICP47 binds human TAP1 with very high affinity, it interacts much less efficiently with murine TAP1, and thus, MHC class I transport is not as efficiently interrupted in mice [142]. Therefore, the murine model of HSV infection may not be as susceptible to NK cell-mediated lysis as would be expected in a human patient. Indeed, there is evidence to suggest that NK cells are important in the innate response against HSV in humans. For instance, patients with severe infections with HSV, including newborns and those with Wiscott-Aldrich syndrome, have low NK cell activity [143]. In addition, the only detectable defect in the immune response of a patient suffering from recurrent severe herpes virus infections was a lack of NK cell activity [144]. More recently, Dalloul *et al.* showed that for two patients with unusually severe HSV infections, pDCs and NK cells could not be detected in the blood and NK cells could not be generated in culture [145]. They suggested that this phenotype is due to the absence of NK cell function, since type I IFN was detected in the serum. Therefore, a role for NK cells in the defense against HSV in humans exists; however, their contribution to innate anti-HSV immunity may be underappreciated in mouse models of infection.

### 4.2. pDCs

pDCs are a functionally distinct subset of DCs whose predominant effector function is the production of type I IFNs. pDCs constitutively express IRF-7, which is involved in the amplification phase of IFN production, and are known to make large amounts of IFNα in response to infection with HSV-1, both in mouse models and in humans [93,146,147]. These cells are also thought to be poor presenters of antigen and are considered professional IFN producing cells (pIPCs).

Given the importance of type I IFN in the innate response to HSV, one would predict that pDCs would be implicated in the defense against this virus. However, the function of these cells in the context of an HSV infection has not been well studied. pDCs were first described in 1999 by Siegal *et al.* as natural interferon producing cells in response to HSV exposure [147]. It was subsequently reported in a mouse model of genital infection that pDCs can recognize HSV-2 exclusively via TLR9 [14]. In fact, mice deficient in either pDCs or TLR9 show similar survival curves when infected with HSV-2 [60]. However, others have shown that in TLR9-deficient mice, pDCs from some tissue sources are capable of limited production of IFNα in response to HSV-1, and thus a minor alternative pathway for HSV detection must exist [148]. In support of a role for pDCs in the defense against HSV, severe inflammation and tissue destruction was seen in pDC deficient mice after genital infection with HSV-2, although mice deficient in TLR9 showed more profound tissue damage [60]. With respect to humans, patients with atopic dermatitis have been found to have decreased numbers of pDCs in the epidermis, which might account for their increased susceptibility to HSV [149]. In addition, an association between low pDC counts and severe recurrent infections with HSV in human patients has been recently proposed [150]. Although pDCs do not support a productive infection by HSV-2, they infiltrate into recurrent HSV genital lesions in human patients; however, their ability to act as APCs remains controversial [151]. Taken together, these data suggest that pDCs may play significant roles in the innate and adaptive response to HSV infection, although further study is required to clarify the function of these cells in antiviral defense.

## 5. Adaptive immune mechanisms against HSV

While innate immunity is thought to play a paramount role in the resistance to HSV, the adaptive immune mechanisms mediating antigen-specific responses cannot be overlooked. Neutralizing antibodies are produced against HSV following infection in both mice and humans, despite playing a seemingly minor role in pathogenesis and immune protection. While earlier studies have elucidated a role for antibody-mediated protection against infection, a growing body of literature highlights the crucial role of cellular immunity, particularly CD8+ T cell-mediated immunity, against HSV. Specifically, the IFNγ-mediated effector functions of CD8+ T cells have been well described and contribute to survival against challenge, maintenance of latency, and limiting of viral spread. In addition, a role for the type I IFNs in CD8+ T cell-mediated immunity highlights the close interaction between the innate and adaptive arms of the immune response against HSV.

### 5.1. Humoral immunity against HSV

While infection with HSV results in the production of neutralizing antibodies, the role of the humoral response in the control of pathogenesis and spread is controversial. On one hand, antibody levels do not determine the outcome of HSV infection in humans. Infants with passive immunity are still capable of being infected and neutralizing antibodies fail to provide sterilizing immunity against infection [152–154]. Passive transfer of immune serum from immunized mice has shown no appreciable effect on viral replication in either the genital tract or the nervous system, implying a nonessential role in both the local control of infection as well as the establishment of latency in the nervous system [16].

On the other hand, early studies in mouse models of HSV-2 have identified a functional role for antibody-mediated protection against transmission. Studies by Zeilin *et al.* and Sherwood *et al.* have shown that administration of anti-HSV antibodies leads to protection against HSV-2 transmission in a vaginal model of infection [155,156]. In these studies, antibody-treated mice have a reduced frequency of clinical disease and/or reduced viral titers at the site of infection. These studies are supported by more recent evidence demonstrating that intraperitoneal administration of a gD-specific antibody reduced the viral load in the vaginal epithelium and protected against the onset of disease [157]. Despite these findings in mice, a lack of clinical verification in humans highlights the need for a better understanding of humoral contributions to HSV control.

The humoral response against HSV has also been associated with long-term effects. Milligan *et al.* demonstrated a sustained detection of HSV-2-specific plasma cells in the bone marrow and spinal cords of infected mice and guinea pigs following vaginal inoculation [158]. Whether antibody levels are strongly correlated with disease severity or reactivation from latency has not been well established. However, evidence from patients with recurring HSV-2 infections demonstrates that IgG1 and IgG3 levels correlate with recurrence of outbreaks [17]. Accordingly, HSV encoded countermeasures against FcγR provide support for antibody-mediated protection [159,160].

Studies in B cell deficient mice also support a role for humoral immunity in immune protection. While μMT B cell deficient mice were able to control replication in the genital mucosa, they were not completely protected against disease [16]. In cyclophosphamide-induced immunosuppressed mice, passive transfer of HSV-1 antiserum led to protection in 100% of mice from mortality, whereas none of the mice receiving control serum survived the challenge, providing strong evidence for a role for antibody-mediated protection against disease progression in the absence of other immune effectors [161]. In an ocular model of HSV-1 infection, it was observed that B cell deficient mice are more susceptible to herpetic encephalitis and keratitis, have increased viral persistence in the eye and increased dissemination to the corneal stroma [162]. As these mice are deficient in T cell mediated immune responses, they generate minimal Th2 responses such as IL4 and IL10 and have diminished proliferative capacity in response to HSV antigen stimulation. Therefore, the role of humoral immunity extends to a role in regulating T cell mediated responses to HSV that correlate with disease severity. Consistent with this observation, Dudley *et al.* found that the antibody contribution to HSV-2 vaginal shedding disappeared following T cell depletion, suggesting that humoral immunity requires a cellular component [163].

Taken together, the specific contribution of humoral immunity to HSV control is not clear. While antibodies against HSV can mediate prophylatic protection in mice, B cells are not absolutely required for protection in the context of an acute infection, and likely interact with other immune effectors such as T cells. As such, a better understanding of this area of HSV immunity is warranted.

### 5.2. Cellular immunity against HSV

It has been shown that CD8+ T cells are recruited to HSV-2 lesions and that infiltration of this cell type occurs early in infection, contributing heavily to immune control and cytolysis. In a cutaneous murine model of HSV-1 infection, proliferation of gB-specific CD8+ T cells occurred less than 48 hours post infection and correlated with cytolytic activity [164]. gB is an immunodominant epitope against which CD8+ T cells are produced; however, gB-nonspecific CD8+ T cells have been identified that have a similar phenotype to gB-specific CD8+ T cells with respect to surface markers, cytokine production, and lysis [165]. This observation suggests that subdominant epitopes contribute equally to the CD8+ T cell response. Not surprisingly, the CD8+ T cell response in patients with genital herpes has been shown to be broadly reactive, with no evidence that responses against specific viral epitopes correlate with outcome [166].

The antiviral effects of CD8+ T cells have been well described, with IFNγ playing a crucial role in this process. For example, neutralizing antibodies against IFNγ lead to impaired resolution of HSV-2 infection [19–22]. In a model of genital HSV-2 infection, IFNγ produced by T cells and not NK cells was required for survival against challenge, although this cytokine is made by both cell types [21]. The cellular response against a TK-negative strain of HSV-2 led to viral control in an intravaginal model of infection in an IFNγ-dependent manner [19]. Not surprisingly, IFNγR^–/–^ mice show significant mortality in response to HSV-1 [167], suggesting a crucial role for this signaling pathway in disease progression.

Given the crucial role of this cell type in the defense against HSV, early establishment of CD8+ T cell responses has been explored as a means of preventing establishment of latency; however, this therapeutic strategy has proved less promising in animal models, which establish a latent infection despite lower viral copy numbers in neural sites as well as decreased viral titers at the site of infection [168]. In fact, multiple studies provide evidence that IFNγ-producing CD8+ T cells persist during neuronal latency in areas of T cell infiltration [165,169], suggesting persistent antigen stimulation and T cell activation during latency despite a failure to clear the infection. Consistent with this finding, Posavad *et al.* found that patients with frequently recurring HSV-2 outbreaks displayed long-term persistence of CD8+ T cells with CTL activity and no evidence of T cell exhaustion [170].

HSV-specific CD8+ T cells have been shown to play a role during HSV latency. In an *ex vivo* reactivation model, CD8+ T cells reduce viral spread from latently infected trigeminal ganglion cells to surrounding fibroblasts in a co-culture system [165]. This evidence is supported by Cantin *et al.* [171], who observed no significant differences between wild-type, IFNγ^–/–^, and IFNγR^–/–^ mice with respect to establishment of latency following HSV-1 infection *in vivo*. Rather, these knockout mice exhibit greater viral gene expression in neurons following reactivation compared to wild-type mice, suggesting that IFNγ contributes to viral control following reactivation from latency as opposed to during the latent phase itself.

In addition to a critical role for CD8+ T cell-mediated antiviral activity against HSV, there is evidence supporting a role for CD4+ T cells in controlling infection. T cell depletion studies have shown that both CD4+ and CD8+ T cells contribute to protection against HSV-2 replication in the local mucosa [172]. While CD8+ effectors are generally thought to be the dominant adaptive immune cell type contributing to protection, it has been recently shown that in both CD8-depleted and CD8-deficient mice, CD4+ T cells are sufficient to clear virus from both the local mucosal as well as neural sites, implying a compensatory role for CD4+ T cells in the absence of CD8+ T cell-mediated protection [23]. This CD4+ T cell-mediated clearance does not rely on Fas or perforin activity, suggesting a non-lytic mechanism for CD4+ T cell effector function. Rather, CD4+ T cells from these mice secrete high levels of IFNγ, highlighting the critical role of this cytokine against HSV. Likewise, Iijima *et al.* describe an IFN-γ-mediated role for CD4+ T cells in clearance of HSV-2 from the vaginal mucosa following secondary challenge [20].

A number of studies have suggested that a synergistic interaction exists between the type I IFNs and IFNγ [6,38,173–180]. It has been shown that mice lacking receptors for both type I and type II IFNs experience a dramatic increase in susceptibility to HSV-1 [6,38], while the absence of either receptor alone causes a much lesser increase in susceptibility [38]. As well, treatment with IFNβ plus IFNγ has been shown to generate a strong decrease in viral replication as well as protection from HSV-1 pathogenesis in a mouse ocular model [178]. Finally, mice lacking STAT1, a key factor in type I and II IFN signaling, are profoundly susceptible to infection with HSV-1 [133,181].

A few distinct mechanisms for this synergy have been described. For example, IFNα has been shown to directly act on CD8+ T cells to mediate cross-presentation of antigen, leading to an expansion of antigen specific CD8+ T cells and specific lysis [182,183]. In addition, a recent study by Trilling *et al.* demonstrated that IFNγ acts in part through IRF-1 to control Vaccinia virus replication in mouse cells *in vitro* [184]. Interestingly, the involvement of IFNAR1, but not IFNβ, in IFNγ-mediation antiviral activity, suggests a complex synergy between these innate and adaptive cytokines.

## 6. Conclusion

In conclusion, the immune response to HSV involves multiple mechanisms to recognize viral components and to target and lyse virally infected cells. The innate immune response represents a first line of defense against incoming virus and plays a crucial role in the early control of infection and spread. The molecular mechanisms underlying this response are numerous, overlapping and in some cases redundant, and largely lead to the production of antiviral protection via the type I IFNs. Both NK cells and pDCs play a leading role in innate control of infection. On the adaptive arm, antibody-mediated protection plays a role, albeit controversial, in the response to HSV, with cellular IFNγ-dependent mechanisms playing a dominant role in control of spread. Finally, the complex interplay between innate and adaptive cytokines demonstrates the necessity of both arms of immunity against infection.

However, multiple lines of conflicting evidence underscore a need for more accurate animal models of HSV that accurately represent the course of infection *in vivo*. In addition, the molecular mechanisms that govern viral recognition still need to be fully elucidated. Finally, manipulating these immune mechanisms into a strategic therapeutic or preventative vaccine against HSV is ongoing and requires further study.

## References

[1] Ellermann-Eriksen S, Liberto M, Iannello D, Mogensen S X-linkage of the early *in vitro* alpha/beta interferon response of mouse peritoneal macrophages to herpes simplex virus type 2. J Gen Virol.

[2] Gresser I, Tovey M, Maury C, Bandu M (1976). Role of interferon in the pathogenesis of virus diseases in mice as demonstrated by the use of anti-interferon serum. II. Studies with herpes simplex, Moloney sarcoma, vesicular stomatitis, Newcastle disease, and influenza viruses. J Exp Med.

[3] Lopez C (1975). Genetics of natural resistance to herpesvirus infections in mice. Nature.

[4] Pedersen E, Haahr S, Mogensen S (1983). X-linked resistance of mice to high doses of herpes simplex virus type 2 correlates with early interferon production. Infect Immun.

[5] Shupack J, Stiller M, Davis I, Kenny C, Jondreau L (1992). Topical alpha-interferon ointment with dimethyl sulfoxide in the treatment of recurrent genital herpes simplex. Dermatology.

[6] Vollstedt S, Arnold S, Schwerdel C, Franchini M, Alber G, Di Santo J, Ackermann M, Suter M (2004). Interplay between alpha/beta and gamma interferons with B, T, and natural killer cells in the defense against herpes simplex virus type 1. J Virol.

[7] Zawatzky R, Engler H, Kirchner H (1982). Experimental infection of inbred mice with herpes simplex virus. III. Comparison between newborn and adult C57BL/6 mice. J Gen Virol.

[8] Zawatzky R, Gresser I, DeMaeyer E, Kirchner H (1982). The role of interferon in the resistance of C57BL/6 mice to various doses of herpes simplex virus type 1. J Infect Dis.

[9] Zawatzky R, Kirchner H, DeMaeyer-Guignard J, DeMaeyer E (1982). An X-linked locus influences the amount of circulating interferon induced in the mouse by herpes simplex virus type 1. J Gen Virol.

[10] Biron C, Brossay L (2001). NK cells and NKT cells in innate defense against viral infections. Curr Opin Immunol.

[11] Cerwenka A, Lanier L (2001). Ligands for natural killer cell receptors: redundancy or specificity. Immunol Rev.

[12] Cooper M, Fehniger T, Caligiuri M (2001). The biology of human natural killer-cell subsets. Trends Immunol.

[13] Kurago Z, Lutz C, Smith K, Colonna M (1998). NK cell natural cytotoxicity and IFN-gamma production are not always coordinately regulated: engagement of DX9 KIR+ NK cells by HLA-B7 variants and target cells. J Immunol.

[14] Lund J, Sato A, Akira S, Medzhitov R, Iwasaki A (2003). Toll-like receptor 9-mediated recognition of Herpes simplex virus-2 by plasmacytoid dendritic cells. J Exp Med.

[15] Milligan GN, Bernstein DI (1995). Generation of humoral immune responses against herpes simplex virus type 2 in the murine female genital tract. Virology.

[16] Morrison LA, Zhu L, Thebeau LG (2001). Vaccine-induced serum immunoglobin contributes to protection from herpes simplex virus type 2 genital infection in the presence of immune T cells. J Virol.

[17] Seppanen M, Meri S, Notkola I-L, Seppala IJT, Hiltunen-Back E, Sarvas H, Lappalainen M, Valimaa H, Palikhe A, Valtonen VV, Lokki M-L (2006). Subtly impaired humoral immunity predisposes to frequently recurring genital herpes simplex virus type 2 infection and herpetic neuralgia. J Infect Dis.

[18] Rouse BT, Gierynska M (2001). Immunity to herpes simplex virus: a hypothesis. Herpes: the journal of the IHMF.

[19] Dobbs ME, Strasser JE, Chu C-F, Chalk C, Milligan GN (2005). Clearance of herpes simplex virus type 2 by CD8+ T cells requires gamma interferon and either perforin- or Fas-mediated cytolytic mechanisms. J Virol.

[20] Iijima N, Linehan MM, Zamora M, Butkus D, Dunn R, Kehry MR, Laufer TM, Iwasaki A (2008). Dendritic cells and B cells maximize mucosal Th1 memory response to herpes simplex virus. J Exp Med.

[21] Milligan GN, Bernstein DI (1997). Interferon-gamma enhances resolution of herpes simplex virus type 2 infection of the murine genital tract. Virology.

[22] Milligan GN, Dudley-McClain KL, Young CG, Chu C-F (2004). T-cell-mediated mechanisms involved in resolution of genital herpes simplex virus type 2 (HSV-2) infection of mice. J Reprod Immunol.

[23] Johnson AJ, Chu C-F, Milligan GN (2008). Effector CD4+ T-cell involvement in clearance of infectious herpes simplex virus type 1 from sensory ganglia and spinal cords. J Virol.

[24] Ellermann-Eriksen S, Justesen J, Mogensen S (1986). Genetically determined difference in the antiviral action of alpha/beta interferon in cells from mice resistant or susceptible to herpes simplex virus type 2. J Gen Virol.

[25] Stulting R, Kindle J, Nahmias A (1985). Patterns of herpes simplex keratitis in inbred mice. Invest Ophthalmol Vis Sci.

[26] Pepose J, Whittum-Hudson J (1987). An immunogenetic analysis of resistance to herpes simplex virus retinitis in inbred strains of mice. Invest Ophthalmol Vis Sci.

[27] Abghari S, Stulting R, Nigida S, Downer D, Nahmias A (1986). Comparative replication of HSV-1 in BALB/c and C57BL/6 mouse embryo fibroblasts *in vitro*. Invest Ophthalmol Vis Sci.

[28] Kastrukoff L, Lau A, Kim S (1987). Multifocal CNS demyelination following peripheral inoculation with herpes simplex virus type 1. Ann Neurol.

[29] Vahlne A, Nilheden E, Svennerholm B (1981). Multiplicity activation of herpes simplex virus in mouse neuroblastoma (C1300) cells. Arch Virol.

[30] Thomas E, Lau A, Kim S, Osborne D, Kastrukoff L (1991). Variation in resistance to herpes simplex virus type 1 of oligodendrocytes derived from inbred strains of mice. J Gen Virol.

[31] Abghari S, Stulting R, Zhu Z, Schinazi R, Kaufman H (1994). Effect of genetically determined host factors on the efficacy of vidarabine, acyclovir and 5-trifluorothymidine in herpes simplex virus type 1 infection. Ophthalmic Res.

[32] Peterson S, Kurimasa A, Oshimura M, Dynan W, Bradbury E, Chen D (1995). Loss of the catalytic subunit of the DNA-dependent protein kinase in DNA double-strand-break-repair mutant mammalian cells. Proc Natl Acad Sci U S A.

[33] Halford WP, Balliet JW, Gebhardt BM (2004). Re-evaluating natural resistance to herpes simplex virus type 1. J Virol.

[34] Murphy J, Duerst R, Smith T, Morrison L (2003). Herpes simplex virus type 2 virion host shutoff protein regulates alpha/beta interferon but not adaptive immune responses during primary infection in vivo. J Virol.

[35] Gill N, Deacon P, Lichty B, Mossman K, Ashkar A (2006). Induction of innate immunity against herpes simplex virus type 2 infection via local delivery of Toll-like receptor ligands correlates with beta interferon production. J Virol.

[36] Svensson A, Bellner L, Magnusson M, Eriksson K (2007). Role of IFN-alpha/beta signaling in the prevention of genital herpes virus type 2 infection. J Reprod Immunol.

[37] Leib D, Harrison T, Laslo K, Machalek M, Moorman N, Virgin H (1999). Interferons regulate the phenotype of wild-type and mutant herpes simplex viruses in vivo. J Exp Med.

[38] Luker G, Prior J, Song J, Pica C, Leib D (2003). Bioluminescence imaging reveals systemic dissemination of herpes simplex virus type 1 in the absence of interferon receptors. J Virol.

[39] Casrouge A, Zhang S, Eidenschenk C, Jouanguy E, Puel A, Yang K, Alcais A, Picard C, Mahfoufi N, Nicolas N, Lorenzo L, Plancoulaine S, Senechal B, Geissmann F, Tabeta K, Hoebe K, Du X, Miller R, Heron B, Mignot C, de Villemeur T, Lebon P, Dulac O, Rozenberg F, Beutler B, Tardieu M, Abel L, Casanova J (2006). Herpes simplex virus encephalitis in human UNC-93B deficiency. Science.

[40] Dupuis S, Jouanguy E, Al-Hajjar S, Fieschi C, Al-Mohsen I, Al-Jumaah S, Yang K, Chapgier A, Eidenschenk C, Eid P, Al Ghonaium A, Tufenkeji H, Frayha H, Al-Gazlan S, Al-Rayes H, Schreiber R, Gresser I, Casanova J (2003). Impaired response to interferon-alpha/beta and lethal viral disease in human STAT1 deficiency. Nat Genet.

[41] Zhang S, Jouanguy E, Ugolini S, Smahi A, Elain G, Romero P, Segal D, Sancho-Shimizu V, Lorenzo L, Puel A, Picard C, Chapgier A, Plancoulaine S, Titeux M, Cognet C, von Bernuth H, Ku C, Casrouge A, Zhang X, Barreiro L, Leonard J, Hamilton C, Lebon P, Heron B, Vallee L, Quintana-Murci L, Hovnanian A, Rozenberg F, Vivier E, Geissmann F, Tardieu M, Abel L, Casanova J (2007). TLR3 deficiency in patients with herpes simplex encephalitis. Science.

[42] Alexopoulou L, Holt A, Medzhitov R, Flavell R (2001). Recognition of double-stranded RNA and activation of NF-[kappa]B by Toll-like receptor 3. Nature.

[43] Fernandes-Alnemri T, Yu J-W, Datta P, Wu J, Alnemri ES (2009). AIM2 activates the inflammasome and cell death in response to cytoplasmic DNA. Nature.

[44] Hornung V, Ablasser A, Charrel-Dennis M, Bauernfeind F, Horvath G, Caffrey DR, Latz E, Fitzgerald KA (2009). AIM2 recognizes cytosolic dsDNA and forms a caspase-1-activating inflammasome with ASC. Nature.

[45] Kato H, Takeuchi O, Sato S, Yoneyama M, Yamamoto M, Matsui K, Uematsu S, Jung A, Kawai T, Ishii KJ, Yamaguchi O, Otsu K, Tsujimura T, Koh C-S, Reis e Sousa C, Matsuura Y, Fujita T, Akira S (2006). Differential roles of MDA5 and RIG-I helicases in the recognition of RNA viruses. Nature.

[46] Limmon GV, Arredouani M, McCann KL, Corn Minor RA, Kobzik L, Imani F (2008). Scavenger receptor class-A is a novel cell surface receptor for double-stranded RNA. FASEB J.

[47] Muruve DA, Pétrilli V, Zaiss AK, White LR, Clark SA, Ross PJ, Parks RJ, Tschopp J (2008). The inflammasome recognizes cytosolic microbial and host DNA and triggers an innate immune response. Nature.

[48] Aravalli RN, Hu S, Rowen TN, Palmquist JM, Lokensgard JR (2005). Cutting edge: TLR2-mediated proinflammatory cytokine and chemokine production by microglial cells in response to herpes simplex virus. J Immunol.

[49] Kurt-Jones EA, Chan M, Zhou S, Wang J, Reed G, Bronson R, Arnold MM, Knipe DM, Finberg RW (2004). Herpes simplex virus 1 interaction with Toll-like receptor 2 contributes to lethal encephalitis. Proc Natl Acad Sci USA.

[50] Hemmi H, Takeuchi O, Kawai T, Kaisho T, Sato S, Sanjo H, Matsumoto M, Hoshino K, Wagner H, Takeda K, Akira S (2000). A Toll-like receptor recognizes bacterial DNA. Nature.

[51] Hausmann S, Marq J-B, Tapparel C, Kolakofsky D, Garcin D (2008). RIG-I and dsRNA-induced IFNbeta activation. PLoS ONE.

[52] Kato H, Takeuchi O, Mikamo-Satoh E, Hirai R, Kawai T, Matsushita K, Hiiragi A, Dermody TS, Fujita T, Akira S (2008). Length-dependent recognition of double-stranded ribonucleic acids by retinoic acid-inducible gene-I and melanoma differentiation-associated gene 5. J Exp Med.

[53] Takaoka A, Wang Z, Choi MK, Yanai H, Negishi H, Ban T, Lu Y, Miyagishi M, Kodama T, Honda K, Ohba Y, Taniguchi T (2007). DAI (DLM-1/ZBP1) is a cytosolic DNA sensor and an activator of innate immune response. Nature.

[54] Chiu Y-H, Macmillan JB, Chen ZJ (2009). RNA polymerase III detects cytosolic DNA and induces type I interferons through the RIG-I pathway. Cell.

[55] Wang Z, Choi MK, Ban T, Yanai H, Negishi H, Lu Y, Tamura T, Takaoka A, Nishikura K, Taniguchi T (2008). Regulation of innate immune responses by DAI (DLM-1/ZBP1) and other DNA-sensing molecules. Proc Natl Acad Sci USA.

[56] Rasmussen SB, Jensen SB, Nielsen C, Quartin E, Kato H, Chen ZJ, Silverman RH, Akira S, Paludan SR (2009). Herpes simplex virus infection is sensed by both Toll-like receptors and retinoic acid-inducible gene- like receptors, which synergize to induce type I interferon production. J Gen Virol.

[57] Rasmussen SB, Sørensen LN, Malmgaard L, Ank N, Baines JD, Chen ZJ, Paludan SR (2007). Type I interferon production during herpes simplex virus infection is controlled by cell-type-specific viral recognition through Toll-like receptor 9, the mitochondrial antiviral signaling protein pathway, and novel recognition systems. J Virol.

[58] Cheng G, Zhong J, Chung J, Chisari FV (2007). Double-stranded DNA and double-stranded RNA induce a common antiviral signaling pathway in human cells. Proc Natl Acad Sci USA.

[59] Krug A, Luker GD, Barchet W, Leib DA, Akira S, Colonna M (2004). Herpes simplex virus type 1 activates murine natural interferon-producing cells through toll-like receptor 9. Blood.

[60] Lund J, Linehan M, Iijima N, Iwasaki A (2006). Cutting Edge: Plasmacytoid dendritic cells provide innate immune protection against mucosal viral infection in situ. J Immunol.

[61] Mansur D, Kroon E, Nogueira M, Arantes R, Rodrigues S, Akira S, Gazzinelli R, Campos M (2005). Lethal encephalitis in myeloid differentiation factor 88-deficient mice infected with herpes simplex virus 1. Am J Pathol.

[62] Wuest T, Austin B, Uematsu S, Thapa M, Akira S, Carr D (2006). Intact TRL 9 and type I interferon signaling pathways are required to augment HSV-1 induced corneal CXCL9 and CXCL10. J Neuroimmunol.

[63] Ashkar A, Rosenthal K (2003). Interleukin-15 and natural killer and NKT cells play a critical role in innate protection against genital herpes simplex virus type 2 infection. J Virol.

[64] Ashkar A, Yao X, Gill N, Sajic D, Patrick A, Rosenthal K (2004). Toll-like receptor (TLR)-3, but not TLR4, agonist protects against genital herpes infection in the absence of inflammation seen with CpG DNA. J Infect Dis.

[65] Harandi A, Eriksson K, Holmgren J (2003). A protective role of locally administered immunostimulatory CpG oligodeoxynucleotide in a mouse model of genital herpes infection. J Virol.

[66] Herbst-Kralovetz M, Pyles R (2006). Quantification of poly(I:C)-mediated protection against genital herpes simplex virus type 2 infection. J Virol.

[67] Pyles R, Higgins D, Chalk C, Zalar A, Eiden J, Brown C, Van Nest G, Stanberry L (2002). Use of immunostimulatory sequence-containing oligonucleotides as topical therapy for genital herpes simplex virus type 2 infection. J Virol.

[68] Sajic D, Ashkar A, Patrick A, McCluskie M, Davis H, Levine K, Holl R, Rosenthal K (2003). Parameters of CpG oligodeoxynucleotide-induced protection against intravaginal HSV-2 challenge. J Med Virol.

[69] Boivin N, Sergerie Y, Rivest S, Boivin G (2008). Effect of pretreatment with toll-like receptor agonists in a mouse model of herpes simplex virus type 1 encephalitis. J Infect Dis.

[70] Boyle K, Pietropaolo R, Compton T (1999). Engagement of the Cellular Receptor for Glycoprotein B of Human Cytomegalovirus Activates the Interferon-Responsive Pathway. Mol. Cell. Biol..

[71] Collins S, Noyce R, Mossman K (2004). Innate cellular response to virus particle entry requires IRF3 but not virus replication. J Virol.

[72] Netterwald J, Jones T, Britt W, Yang S-J, McCrone I, Zhu H (2004). Postattachment Events Associated with Viral Entry Are Necessary for Induction of Interferon-Stimulated Genes by Human Cytomegalovirus. J. Virol..

[73] Noyce RS, Collins SE, Mossman KL (2009). Differential modification of interferon regulatory factor 3 following virus particle entry. J Virol.

[74] Paladino P, Cummings D, Noyce R, Mossman K (2006). The IFN-independent response to virus particle entry provides a first line of antiviral defense that is independent of TLRs and retinoic acid-inducible gene I. J Immunol.

[75] Prescott J, Ye C, Sen G, Hjelle B (2005). Induction of Innate Immune Response Genes by Sin Nombre Hantavirus Does Not Require Viral Replication. J. Virol..

[76] Preston C, Harman A, Nicholl M (2001). Activation of interferon response factor-3 in human cells infected with herpes simplex virus type 1 or human cytomegalovirus. J Virol.

[77] Duncan JA, Bergstralh DT, Wang Y, Willingham SB, Ye Z, Zimmermann AG, Ting JP-Y (2007). Cryopyrin/NALP3 binds ATP/dATP, is an ATPase, and requires ATP binding to mediate inflammatory signaling. Proc Natl Acad Sci USA.

[78] Mariathasan S, Weiss DS, Newton K, McBride J, O’Rourke K, Roose-Girma M, Lee WP, Weinrauch Y, Monack DM, Dixit VM (2006). Cryopyrin activates the inflammasome in response to toxins and ATP. Nature.

[79] Pétrilli V, Papin S, Dostert C, Mayor A, Martinon F, Tschopp J (2007). Activation of the NALP3 inflammasome is triggered by low intracellular potassium concentration. Cell Death Differ.

[80] Fitzgerald K, McWhirter S, Faia K, Rowe D, Latz E, Golenbock D, Coyle A, Liao S, Maniatis T (2003). IKKepsilon and TBK1 are essential components of the IRF3 signaling pathway. Nat Immunol.

[81] Sharma S, tenOever B, Grandvaux N, Zhou G-P, Lin R, Hiscott J (2003). Triggering the Interferon Antiviral Response Through an IKK-Related Pathway. Science.

[82] Zhong B, Yang Y, Li S, Wang Y-Y, Li Y, Diao F, Lei C, He X, Zhang L, Tien P, Shu H-B (2008). The adaptor protein MITA links virus-sensing receptors to IRF3 transcription factor activation. Immunity.

[83] McWhirter SM, Fitzgerald KA, Rosains J, Rowe DC, Golenbock DT, Maniatis T (2004). IFN-regulatory factor 3-dependent gene expression is defective in Tbk1-deficient mouse embryonic fibroblasts. Proc Natl Acad Sci USA.

[84] Grandvaux N, Servant M, tenOever B, Sen G, Balachandran S, Barber G, Lin R, Hiscott J (2002). Transcriptional Profiling of Interferon Regulatory Factor 3 Target Genes: Direct Involvement in the Regulation of Interferon-Stimulated Genes. J. Virol..

[85] Kumar K, McBride K, Weaver B, Dingwall C, Reich N (2000). Regulated Nuclear-Cytoplasmic Localization of Interferon Regulatory Factor 3, a Subunit of Double-Stranded RNA-Activated Factor 1. Mol. Cell. Biol..

[86] Lin R, Heylbroeck C, Pitha P, Hiscott J (1998). Virus-Dependent Phosphorylation of the IRF-3 Transcription Factor Regulates Nuclear Translocation, Transactivation Potential, and Proteasome-Mediated Degradation. Mol. Cell. Biol..

[87] Qin B, Liu C, Srinath H, Lam S, Correia J, Derynck R, Lin K (2005). Crystal Structure of IRF-3 in Complex with CBP. Structure.

[88] Qin BY, Liu C, Lam SS, Srinath H, Delston R, Correia JJ, Derynck R, Lin K (2003). Crystal structure of IRF-3 reveals mechanism of autoinhibition and virus-induced phosphoactivation. Nat Struct Biol.

[89] Takahasi K, Suzuki NN, Horiuchi M, Mori M, Suhara W, Okabe Y, Fukuhara Y, Terasawa H, Akira S, Fujita T, Inagaki F (2003). X-ray crystal structure of IRF-3 and its functional implications. Nat Struct Biol.

[90] Lin R, Noyce R, Collins S, Everett R, Mossman K (2004). The herpes simplex virus ICP0 RING finger domain inhibits IRF3- and IRF7-mediated activation of interferon-stimulated genes. J Virol.

[91] Melroe G, DeLuca N, Knipe D (2004). Herpes simplex virus 1 has multiple mechanisms for blocking virus-induced interferon production. J Virol.

[92] Melroe G, Silva L, Schaffer P, Knipe D (2007). Recruitment of activated IRF-3 and CBP/p300 to herpes simplex virus ICP0 nuclear foci: Potential role in blocking IFN-beta induction. Virology.

[93] Honda K, Yanai H, Negishi H, Asagiri M, Sato M, Mizutani T, Shimada N, Ohba Y, Takaoka A, Yoshida N, Taniguchi T (2005). IRF-7 is the master regulator of type-I interferon-dependent immune responses. Nature.

[94] D’Cunha J, Knight E, Haas A, Truitt R, Borden E (1996). Immunoregulatory properties of ISG15, an interferon-induced cytokine. PNAS.

[95] Guo J, Peters K, Sen G (2000). Induction of the Human Protein P56 by Interferon, Double-Stranded RNA, or Virus Infection. Virology.

[96] Hui D, Bhasker C, Merrick W, Sen G (2003). Viral Stress-inducible Protein p56 Inhibits Translation by Blocking the Interaction of eIF3 with the Ternary Complex eIF2{middle dot}GTP{middle dot}Met-tRNAi. J. Biol. Chem..

[97] Marques J, Rebouillat D, Ramana C, Murakami J, Hill J, Gudkov A, Silverman R, Stark G, Williams B (2005). Down-Regulation of p53 by Double-Stranded RNA Modulates the Antiviral Response. J. Virol..

[98] Zhou A, Hassel B, Silverman R (1993). Expression cloning of 2–5A-dependent RNAase: A uniquely regulated mediator of interferon action. Cell.

[99] Paladino P, Mossman KL (2009). Mechanisms employed by herpes simplex virus 1 to inhibit the interferon response. J Interferon Cytokine Res.

[100] Lavau C, Marchio A, Fagioli M, Jansen J, Falini B, Lebon P, Grosveld F, Pandolfi PP, Pelicci PG, Dejean A (1995). The acute promyelocytic leukaemia-associated PML gene is induced by interferon. Oncogene.

[101] Wang ZG, Ruggero D, Ronchetti S, Zhong S, Gaboli M, Rivi R, Pandolfi PP (1998). PML is essential for multiple apoptotic pathways. Nat Genet.

[102] Zheng P, Guo Y, Niu Q, Levy DE, Dyck JA, Lu S, Sheiman LA, Liu Y (1998). Proto-oncogene PML controls genes devoted to MHC class I antigen presentation. Nature.

[103] Everett RD, Maul GG (1994). HSV-1 IE protein Vmw110 causes redistribution of PML. EMBO J.

[104] Maul GG, Everett RD (1994). The nuclear location of PML, a cellular member of the C3HC4 zinc-binding domain protein family, is rearranged during herpes simplex virus infection by the C3HC4 viral protein ICP0. J Gen Virol.

[105] Farrell PJ, Sen GC, Dubois MF, Ratner L, Slattery E, Lengyel P (1978). Interferon action: two distinct pathways for inhibition of protein synthesis by double-stranded RNA. Proc Natl Acad Sci USA.

[106] Al-Khatib K, Williams B, Silverman R, Halford W, Carr D (2004). Distinctive roles for 2′,5′-oligoadenylate synthetases and double-stranded RNA-dependent protein kinase R in the in vivo antiviral effect of an adenoviral vector expressing murine IFN-beta. J Immunol.

[107] Carr D, Tomanek L, Silverman R, Campbell I, Williams B (2005). RNA-dependent protein kinase is required for alpha-1 interferon transgene-induced resistance to genital herpes simplex virus type 2. J Virol.

[108] He B, Gross M, Roizman B (1997). The gamma(1)34.5 protein of herpes simplex virus 1 complexes with protein phosphatase 1alpha to dephosphorylate the alpha subunit of the eukaryotic translation initiation factor 2 and preclude the shutoff of protein synthesis by double-stranded RNA-activated protein kinase. Proc Natl Acad Sci USA.

[109] Poppers J, Mulvey M, Khoo D, Mohr I (2000). Inhibition of PKR activation by the proline-rich RNA binding domain of the herpes simplex virus type 1 Us11 protein. J Virol.

[110] Duerst RJ, Morrison LA (2004). Herpes simplex virus 2 virion host shutoff protein interferes with type I interferon production and responsiveness. Virology.

[111] Karlhofer F, Ribaudo R, Yokoyama W (1992). MHC class I alloantigen specificity of Ly-49+ IL-2-activated natural killer cells. Nature.

[112] Wagtmann N, Biassoni R, Cantoni C, Verdiani S, Malnati M, Vitale M, Bottino C, Moretta L, Moretta A, Long E (1995). Molecular clones of the p58 NK cell receptor reveal immunoglobulin-related molecules with diversity in both the extra- and intracellular domains. Immunity.

[113] Mocikat R, Braumuller H, Gumy A, Egeter O, Ziegler H, Reusch U, Bubeck A, Louis J, Mailhammer R, Riethmuller G, Koszinowski U, Rocken M (2003). Natural killer cells activated by MHC class I(low) targets prime dendritic cells to induce protective CD8 T cell responses. Immunity.

[114] York I, Roop C, Andrews D, Riddell S, Graham F, Johnson D (1994). A cytosolic herpes simplex virus protein inhibits antigen presentation to CD8+ T lymphocytes. Cell.

[115] Rola-Pleszczynski M (1981). *In vitro* induction of human cell-mediated cytotoxicity directed against herpes simplex virus-infected cells. Kinetics in normal donors and patients with recurrent herpes labialis. J Clin Lab Immunol.

[116] Bishop G, Glorioso J, Schwartz S (1983). Relationship between expression of herpes simplex virus glycoproteins and susceptibility of target cells to human natural killer activity. J Exp Med.

[117] Fitzgerald P, Mendelsohn M, Lopez C (1985). Human natural killer cells limit replication of herpes simplex virus type 1 *in vitro*. J Immunol.

[118] Smith H, Heusel J, Mehta I, Kim S, Dorner B, Naidenko O, Iizuka K, Furukawa H, Beckman D, Pingel J, Scalzo A, Fremont D, Yokoyama W (2002). Recognition of a virus-encoded ligand by a natural killer cell activation receptor. Proc Natl Acad Sci U S A.

[119] Ghiasi H, Cai S, Perng G, Nesburn A, Wechsler S (2000). The role of natural killer cells in protection of mice against death and corneal scarring following ocular HSV-1 infection. Antiviral Res.

[120] Habu S, Akamatsu K, Tamaoki N, Okumura K (1984). In vivo significance of NK cell on resistance against virus (HSV-1) infections in mice. J Immunol.

[121] Nandakumar S, Woolard S, Yuan D, Rouse B, Kumaraguru U (2008). Natural killer cells as novel helpers in anti-herpes simplex virus immune response. J Virol.

[122] Pereira R, Scalzo A, Simmons A (2001). Cutting edge: a NK complex-linked locus governs acute versus latent herpes simplex virus infection of neurons. J Immunol.

[123] Staats H, Oakes J, Lausch R (1991). Anti-glycoprotein D monoclonal antibody protects against herpes simplex virus type 1-induced diseases in mice functionally depleted of selected T-cell subsets or asialo GM1+ cells. J Virol.

[124] Tanigawa M, Bigger J, Kanter M, Atherton S (2000). Natural killer cells prevent direct anterior-to-posterior spread of herpes simplex virus type 1 in the eye. Invest Ophthalmol Vis Sci.

[125] Thapa M, Kuziel W, Carr D (2007). Susceptibility of CCR5-deficient mice to genital herpes simplex virus type 2 is linked to NK cell mobilization. J Virol.

[126] Bukowski J, Welsh R (1986). The role of natural killer cells and interferon in resistance to acute infection of mice with herpes simplex virus type 1. J Immunol.

[127] Chmielarczyk W, Engler H, Ernst R, Opitz U, Kirchner H (1985). Injection of anti-thy-1.2 serum breaks genetic resistance of mice against herpes simplex virus. J Gen Virol.

[128] Kassim S, Rajasagi N, Ritz B, Pruett S, Gardner E, Chervenak R, Jennings S (2009). Dendritic cells are required for optimal activation of natural killer functions following primary infection with herpes simplex virus type 1. J Virol.

[129] Mercurio A, Schwarting G, Robbins P (1984). Glycolipids of the mouse peritoneal macrophage. Alterations in amount and surface exposure of specific glycolipid species occur in response to inflammation and tumoricidal activation. J Exp Med.

[130] Schuler G (1984). The dendritic, Thy-1-positive cell of murine epidermis: a new epidermal cell type of bone marrow origin. J Invest Dermatol.

[131] Suttles J, Schwarting G, Stout R (1986). Flow cytometric analysis reveals the presence of asialo GM1 on the surface membrane of alloimmune cytotoxic T lymphocytes. J Immunol.

[132] Wiltrout R, Santoni A, Peterson E, Knott D, Overton W, Herberman R, Holden H (1985). Reactivity of anti-asialo GM1 serum with tumoricidal and non-tumoricidal mouse macrophages. J Leukoc Biol.

[133] Halford WP, Maender JL, Gebhardt BM (2005). Re-evaluating the role of natural killer cells in innate resistance to herpes simplex virus type 1. Virol J.

[134] Slifka M, Pagarigan R, Whitton J (2000). NK markers are expressed on a high percentage of virus-specific CD8+ and CD4+ T cells. J Immunol.

[135] Rager-Zisman B, Quan P, Rosner M, Moller J, Bloom B (1987). Role of NK cells in protection of mice against herpes simplex virus-1 infection. J Immunol.

[136] Adler H, Beland J, Del-Pan N, Kobzik L, Sobel R, Rimm I (1999). In the absence of T cells, natural killer cells protect from mortality due to HSV-1 encephalitis. J Neuroimmunol.

[137] Williams N, Klem J, Puzanov I, Sivakumar P, Schatzle J, Bennett M, Kumar V (1998). Natural killer cell differentiation: insights from knockout and transgenic mouse models and *in vitro* systems. Immunol Rev.

[138] Gill N, Rosenthal K, Ashkar A (2005). NK and NKT cell-independent contribution of interleukin-15 to innate protection against mucosal viral infection. J Virol.

[139] Grubor-Bauk B, Simmons A, Mayrhofer G, Speck P (2003). Impaired clearance of herpes simplex virus type 1 from mice lacking CD1d or NKT cells expressing the semivariant V alpha 14-J alpha 281 TCR. J Immunol.

[140] Brandt C, Salkowski C (1992). Activation of NK cells in mice following corneal infection with herpes simplex virus type-1. Invest Ophthalmol Vis Sci.

[141] Gill N, Ashkar A (2009). Overexpression of interleukin-15 compromises CD4-dependent adaptive immune responses against herpes simplex virus 2. J Virol.

[142] Tomazin R, van Schoot N, Goldsmith K, Jugovic P, Sempe P, Fruh K, Johnson D (1998). Herpes simplex virus type 2 ICP47 inhibits human TAP but not mouse TAP. J Virol.

[143] Ching C, Lopez C (1979). Natural killing of herpes simplex virus type 1-infected target cells: normal human responses and influence of antiviral antibody. Infect Immun.

[144] Biron C, Byron K, Sullivan J (1989). Severe herpesvirus infections in an adolescent without natural killer cells. N Engl J Med.

[145] Dalloul A, Oksenhendler E, Chosidow O, Ribaud P, Carcelain G, Louvet S, Massip P, Lebon P, Autran B (2004). Severe herpes virus (HSV-2) infection in two patients with myelodysplasia and undetectable NK cells and plasmacytoid dendritic cells in the blood. J Clin Virol.

[146] McKenna K, Beignon A, Bhardwaj N (2005). Plasmacytoid dendritic cells: linking innate and adaptive immunity. J Virol.

[147] Siegal F, Kadowaki N, Shodell M, Fitzgerald-Bocarsly P, Shah K, Ho S, Antonenko S, Liu Y (1999). The nature of the principal type 1 interferon-producing cells in human blood. Science.

[148] Hochrein H, Schlatter B, O’Keeffe M, Wagner C, Schmitz F, Schiemann M, Bauer S, Suter M, Wagner H (2004). Herpes simplex virus type-1 induces IFN-alpha production via Toll-like receptor 9-dependent and -independent pathways. Proc Natl Acad Sci U S A.

[149] Wollenberg A, Wagner M, Gunther S, Towarowski A, Tuma E, Moderer M, Rothenfusser S, Wetzel S, Endres S, Hartmann G (2002). Plasmacytoid dendritic cells: a new cutaneous dendritic cell subset with distinct role in inflammatory skin diseases. J Invest Dermatol.

[150] Kittan N, Bergua A, Haupt S, Donhauser N, Schuster P, Korn K, Harrer T, Schmidt B (2007). Impaired plasmacytoid dendritic cell innate immune responses in patients with herpes virus-associated acute retinal necrosis. J Immunol.

[151] Donaghy H, Bosnjak L, Harman A, Marsden V, Tyring S, Meng T, Cunningham A (2009). Role for plasmacytoid dendritic cells in the immune control of recurrent human herpes simplex virus infection. J Virol.

[152] Brown ZA, Selke S, Zeh J, Kopelman J, Maslow A, Ashley RL, Watts DH, Berry S, Herd M, Corey L (1997). The acquisition of herpes simplex virus during pregnancy. N Engl J Med.

[153] Kuklin NA, Daheshia M, Chun S, Rouse BT (1998). Role of mucosal immunity in herpes simplex virus infection. J Immunol.

[154] Kohl S, Loo LS (1984). The relative role of transplacental and milk immune transfer in protection against lethal neonatal herpes simplex virus infection in mice. J Infect Dis.

[155] Sherwood JK, Zeitlin L, Whaley KJ, Cone RA, Saltzman M (1996). Controlled release of antibodies for long-term topical passive immunoprotection of female mice against genital herpes. Nat Biotechnol.

[156] Zeitlin L, Whaley KJ, Sanna PP, Moench TR, Bastidas R, De Logu A, Williamson RA, Burton DR, Cone RA (1996). Topically applied human recombinant monoclonal IgG1 antibody and its Fab and F(ab’)2 fragments protect mice from vaginal transmission of HSV-2. Virology.

[157] Chu C-F, Meador MG, Young CG, Strasser JE, Bourne N, Milligan GN (2008). Antibody-mediated protection against genital herpes simplex virus type 2 disease in mice by Fc gamma receptor-dependent and -independent mechanisms. J Reprod Immunol.

[158] Milligan GN, Meador MG, Chu C-F, Young CG, Martin TL, Bourne N (2005). Long-term presence of virus-specific plasma cells in sensory ganglia and spinal cord following intravaginal inoculation of herpes simplex virus type 2. J Virol.

[159] Para MF, Baucke RB, Spear PG (1980). Immunoglobulin G(Fc)-binding receptors on virions of herpes simplex virus type 1 and transfer of these receptors to the cell surface by infection. J Virol.

[160] Baucke RB, Spear PG (1979). Membrane proteins specified by herpes simplex viruses. V. Identification of an Fc-binding glycoprotein. J Virol.

[161] Mitchell BM, Stevens JG (1996). Neuroinvasive properties of herpes simplex virus type 1 glycoprotein variants are controlled by the immune response. J Immunol.

[162] Deshpande SP, Zheng M, Daheshia M, Rouse BT (2000). Pathogenesis of herpes simplex virus-induced ocular immunoinflammatory lesions in B-cell-deficient mice. J Virol.

[163] Dudley KL, Bourne N, Milligan GN (2000). Immune protection against HSV-2 in B-cell-deficient mice. Virology.

[164] Mueller SN, Jones CM, Smith CM, Heath WR, Carbone FR (2002). Rapid cytotoxic T lymphocyte activation occurs in the draining lymph nodes after cutaneous herpes simplex virus infection as a result of early antigen presentation and not the presence of virus. J Exp Med.

[165] Sheridan BS, Cherpes TL, Urban J, Kalinski P, Hendricks RL (2009). Reevaluating the CD8 T-cell response to herpes simplex virus type 1: involvement of CD8 T cells reactive to subdominant epitopes. J Virol.

[166] Hosken N, McGowan P, Meier A, Koelle DM, Sleath P, Wagener F, Elliott M, Grabstein K, Posavad C, Corey L (2006). Diversity of the CD8+ T-cell response to herpes simplex virus type 2 proteins among persons with genital herpes. J Virol.

[167] Cantin E, Tanamachi B, Openshaw H, Mann J, Clarke K (1999). Gamma interferon (IFN-gamma) receptor null-mutant mice are more susceptible to herpes simplex virus type 1 infection than IFN-gamma ligand null-mutant mice. J Virol.

[168] Wakim LM, Jones CM, Gebhardt T, Preston CM, Carbone FR (2008). CD8(+) T-cell attenuation of cutaneous herpes simplex virus infection reduces the average viral copy number of the ensuing latent infection. Immunol Cell Biol.

[169] Cantin E, Hinton D, Chen J, Openshaw H (1995). Gamma interferon expression during acute and latent nervous system infection by herpes simplex virus type 1. J Virol.

[170] Posavad CM, Huang ML, Barcy S, Koelle DM, Corey L (2000). Long term persistence of herpes simplex virus-specific CD8+ CTL in persons with frequently recurring genital herpes. J Immunol.

[171] Cantin E, Tanamachi B, Openshaw H (1999). Role for gamma interferon in control of herpes simplex virus type 1 reactivation. J Virol.

[172] Milligan GN, Bernstein DI, Bourne N (1998). T lymphocytes are required for protection of the vaginal mucosae and sensory ganglia of immune mice against reinfection with herpes simplex virus type 2. J Immunol.

[173] Balish M, Abrams M, Pumfery A, Brandt C (1992). Enhanced inhibition of herpes simplex virus type 1 growth in human corneal fibroblasts by combinations of interferon-alpha and -gamma. J Infect Dis.

[174] Chen S, Oakes J, Lausch R (1993). Synergistic anti-HSV effect of tumor necrosis factor alpha and interferon gamma in human corneal fibroblasts is associated with interferon beta induction. Antiviral Res.

[175] Czarniecki C, Fennie C, Powers D, Estell D (1984). Synergistic antiviral and antiproliferative activities of Escherichia coli-derived human alpha, beta, and gamma interferons. J Virol.

[176] Neumann-Haefelin D, Sundmacher R, Frey H, Merk W (1985). Recombinant HuIFN-gamma prevents herpes simplex keratitis in African green monkeys: demonstration of synergism with recombinant HuIFN-alpha 2. Med Microbiol Immunol.

[177] Zerial A, Hovanessian A, Stefanos S, Huygen K, Werner G, Falcoff E (1982). Synergistic activities of type I (alpha, beta) and type II (gamma) murine interferons. Antiviral Res.

[178] Sainz B, Halford W (2002). Alpha/Beta interferon and gamma interferon synergize to inhibit the replication of herpes simplex virus type 1. J Virol.

[179] Chee A, Lopez P, Pandolfi P, Roizman B (2003). Promyelocytic leukemia protein mediates interferon-based anti-herpes simplex virus 1 effects. J Virol.

[180] Pierce A, DeSalvo J, Foster T, Kosinski A, Weller S, Halford W (2005). Beta interferon and gamma interferon synergize to block viral DNA and virion synthesis in herpes simplex virus-infected cells. J Gen Virol.

[181] Pasieka T, Cilloniz C, Lu B, Teal T, Proll S, Katze M, Leib D (2009). Host responses to wild-type and attenuated herpes simplex virus infection in the absence of Stat1. J Virol.

[182] Le Bon A, Durand V, Kamphuis E, Thompson C, Bulfone-Paus S, Rossmann C, Kalinke U, Tough D (2006). Direct stimulation of T cells by type I IFN enhances the CD8+ T cell response during cross-priming. J Immunol.

[183] Le Bon A, Etchart N, Rossmann C, Ashton M, Hou S, Gewert D, Borrow P, Tough DF (2003). Cross-priming of CD8+ T cells stimulated by virus-induced type I interferon. Nat Immunol.

[184] Trilling M, Le VTK, Zimmermann A, Ludwig H, Pfeffer K, Sutter G, Smith GL, Hengel H (2009). Gamma interferon-induced interferon regulatory factor 1-dependent antiviral response inhibits vaccinia virus replication in mouse but not human fibroblasts. J Virol.

